# Speech recognition performance with dual-microphone audio processors in mandarin-speaking cochlear implant users

**DOI:** 10.3389/fnins.2026.1767325

**Published:** 2026-04-23

**Authors:** Kailong Yin, Shuo Chen, Fei Wang, Lian Hui

**Affiliations:** Department of Otolaryngology, The First Hospital of China Medical University, Shenyang, China

**Keywords:** adaptive noise reduction, audio processors, cochlear implants, mandarin Chinese users, speech recognition in noise

## Abstract

**Background:**

Cochlear implant (CI) audio processor upgrades have demonstrated speech recognition benefits in non-tonal language populations, but high-level evidence for native Mandarin-speaking CI users (a tonal language with unique signal processing requirements) remains critically limited. This study aimed to assess the speech perception performance of the SONNET 2 and RONDO 3 dual-microphone audio processors in native Mandarin-speaking CI users.

**Methods:**

This prospective single-subject repeated-measures study enrolled 51 native Mandarin-speaking CI users. Speech recognition performance was tested across five processor configurations: the legacy baseline processor, SONNET 2 (S2) in omnidirectional (S2.OMNI) and adaptive intelligence (S2.Adaptive) modes, and RONDO 3 (R3) in omnidirectional (R3.OMNI) and adaptive intelligence (R3.Adaptive) modes. Outcome measures included monosyllabic words, disyllabic words, and sentence recognition in quiet, and sentence recognition in co-located speech-shaped noise (S0N0 paradigm). The pre-specified primary endpoint was sentence recognition in noise for S2.OMNI vs. the legacy processor; confirmatory linear mixed-effects models (LMMs) and subgroup analyses were exclusively performed for the primary endpoint, with pairwise comparisons for all secondary exploratory endpoints.

**Results:**

In quiet, all four upgraded configurations yielded significantly higher monosyllabic word recognition scores vs. the legacy baseline (all *p* < 0.05, FDR-adjusted); all configurations except R3. Adaptive showed significant improvements in disyllabic word recognition (all *p* < 0.05, FDR-adjusted). In the S0N0 noise condition, S2.OMNI and R3.OMNI significantly enhanced sentence recognition vs. the legacy processor (*p* < 0.001 and *p* = 0.011, respectively, FDR-adjusted), while no significant benefit was detected for either adaptive mode after FDR correction. LMM analysis confirmed that upgrading to the S2.OMNI configuration was an independent positive predictor of noise sentence recognition (*F* = 9.885, *p* = 0.003), with consistent significant benefits across pediatric/adult and unilateral/bilateral users in pre-specified subgroup analyses.

**Conclusion:**

This study provides confirmatory evidence that upgrading to the S2.OMNI configuration significantly improves sentence recognition in noise in native Mandarin-speaking CI users, with consistent benefits across key clinical subgroups. Exploratory analyses show that the S2 and R3 processors also deliver significant improvements in word recognition in quiet, These results fill a critical evidence gap for tonal language CI populations and may help guide clinical device selection.

## 1 Introduction

Cochlear implant (CI) use is the most effective treatment for individuals with severe-to-profound sensorineural hearing loss, and over 1 million individuals have been implanted (Zeng, 2022; Schvartz-Leyzac et al., 2023). Most CI users achieve good speech recognition in quiet environments (McGregor and Goldman, 2022), but hearing in background noise remains especially challenging. (Henry et al., 2023; Kurz et al., 2022; Thai et al., 2022) Consequently, improving speech recognition in noisy environments remains a key research focus (Dhanasingh, 2021; Mosnier et al., 2021; Büchner and Gärtner, 2017; Hey et al., 2021).

For audio processor signal processing, there are two broad approaches to reduce the impact of noise on speech recognition. The first is noise reduction algorithms which attempt to model and suppress background noise in the signal. These algorithms have demonstrated significant benefits but work best for stationary noise environments (Stronks et al., 2021; Dawson et al., 2011). The other approach is the use of directional microphone systems (Hersbach et al., 2012; Fischer et al., 2021; Sivonen et al., 2020; Goffi-Gomez et al., 2021; Jespersen et al., 2021), where the signals from an array of microphones are compared and the signal from the direction of maximum (non-noise) is selectively enhanced to improve the signal-to-noise ratio (SNR) (Warren et al., 2019). These systems improve speech recognition in noise but work best when target speech and noise are spatially separated (Weissgerber et al., 2019; Carlyon and Goehring, 2021; Kuk et al., 2023).

The audio processors used in the present investigation were the SONNET2 (S2) and the RONDO3 (R3) (both from MED-EL, Innsbruck, Austria). Both feature dual microphone arrays to facilitate microphone directionality (MD) via beamforming as well as adaptive noise reduction (“*adaptive intelligence*”). The latter system classifies the acoustic environment into five categories (quiet, speech, speech in noise, noise, and music) and automatically selects the optimal combination of processing settings for ambient noise reduction (ANR), transient noise reduction (TNR), wind noise reduction (WNR), and MD for each sound category (Buechner et al., 2014). The S2 has a behind-the-ear (BTE) configuration while the R3 has an off-the-ear (OTE) configuration. The R3 also has a built-in lithium battery for wireless charging, which eliminates accessory needs and drying requirements, thus reducing costs for the user (Mauger et al., 2017).

Prior studies have evaluated various aspects of these noise reduction methods in CI users. Kurz et al. evaluated speech perception in 20 adult unilateral S2 users across different speech-noise spatial configurations and observed that adaptive noise reduction significantly improved speech recognition in noise (Kurz et al., 2022). Hagen evaluated the performance of 31 adult unilateral CI users in quiet and in the presence of simulated wind noise at different speech-noise spatial configurations. They observed that MD improved speech understanding in simulated wind noise under acoustic conditions where target speech and interfering noise were spatially separated (Hagen et al., 2020). Honeder et al. (2018) measured speech reception thresholds in 18 post-lingually deafened adult experienced CI users. Performance was measured using omnidirectional, directional, and adaptive beamforming microphones with noise speakers positioned at −135° and 135°. They observed that the adaptive microphone mode improved speech recognition in noise.

Existing research on speech recognition for CI users predominantly focuses on non-tonal languages. In Mandarin Chinese, lexical meaning depends on pitch contours (for example /ma^1^/ 妈 “mother” vs. /ma^3^/ 马 “horse”). For these languages, speech recognition in noise may be even more challenging. Our study evaluated speech recognition abilities in Mandarin-speaking adult and pediatric CI users (a typical tonal language population) using the S2 and R3 audio processors, both in quiet and in various background noise conditions.

## 2 Materials and methods

### 2.1 Ethics, informed consent, and inclusion criteria

This study was approved by The Ethics Committee of The First Hospital of China Medical University (approval number: 2024-963-2). All participants provided written informed consent prior to participation.

The inclusion criteria were a diagnosis of bilateral severe or profound sensorineural hearing loss (WHO 1997 criteria), ≥6 months of CI experience, native Mandarin Chinese speaker, and age ≥6 years.

### 2.2 Study participants

51 participants (27 unilateral, 24 bilateral) were included, providing data from 75 ears. For bilateral recipients, only data from the earlier-implanted ear were used in all analyses. For participants who received simultaneous bilateral implantation, the average score of both ears was used. Thus, all statistical analyses were performed at the participant level (*n* = 51). The mean age (±SD) at the beginning of the study was 19.98 (±16.06) years (range: 6–75 years). The mean age (±SD) at surgery was 12.59 (±18.02) years (range: 1–72 years). Thirty participants were children (6–16 years) and 21 were adults (>16 years). All had undergone unilateral (*n* = 27) or bilateral (*n* = 24) cochlear implantation. Some wore a hearing aid on the contralateral side. The implant models were the Combi40+ (*n* = 9), CONCERTO (*n* = 20), PULSAR (*n* = 1), SYNCHRONY (*n* = 9), and SONATA (*n* = 36).

### 2.3 Study design

This prospective study employed a single-subject repeated-measures design, with all experimental procedures conducted in a sound-controlled acute testing environment. All participants were blinded to the model and operating mode of the audio processor throughout the entire testing session. For all assessments, unilateral testing was performed exclusively: contralateral amplification devices (hearing aids or contralateral CI audio processors) were removed prior to testing, and complete masking of the non-test contralateral ear was achieved via ER3A insert earphones delivering 60 dB broadband white noise, in conjunction with double-layer sound-attenuating earmuffs (3 M Peltor X5A, 33 dB noise reduction rating [NRR]).

Five audio processor configurations were tested:

- The legacy audio processor (as listed above in 2.2).
- The S2 using either omnidirectional mode (S2.OMNI) or the adaptive intelligence mode (S2.Adaptive).
- The R3 using either omnidirectional mode (R3.OMNI) or the adaptive intelligence mode (R3.Adaptive).

For each configuration, speech recognition scores for monosyllables, disyllables, sentence recognition in quiet, and sentence recognition in noise were measured.

All behavioral speech assessments were conducted in a double-walled, sound-attenuating booth, using a Conera audiometer (Otometrics, Denmark) interfaced with a dedicated test computer. Two loudspeakers were positioned at a distance of 1 m directly in front of the participant (0° azimuth in the horizontal plane): the first loudspeaker delivered speech stimuli at a fixed presentation level of 70 dB sound pressure level (SPL), while the second delivered background noise at a fixed level of 60 dB SPL.

Speech recognition testing in quiet was performed using validated Mandarin speech assessment batteries, with age-appropriate materials selected for each participant. For adult participants, the Mandarin Speech Test Materials (MSTMs) were used. The MSTM battery comprises of recorded speech stimuli produced by a native male adult Mandarin speaker. To mitigate learning bias associated with word memorization, unique word lists were randomly assigned by the test system for each assessment, with no list reuse across the entire study cohort. For pediatric participants, the Mandarin Pediatric Speech Intelligibility (MPSI) test was used for quiet-environment speech assessments. Speech-in-noise testing was conducted using the Mandarin Hearing in Noise Test (MHINT), with standard speech-shaped noise as the background masker. The MHINT battery includes 12 validated test lists, each consisting of 20 short sentences (10 characters per sentence).

For all participants, the audio processor’s adaptive intelligence algorithm was configured with the manufacturer’s default factory preset (AI Mild), with no manual parameter adjustments during testing. Loudness balancing and comfort threshold checks were performed for each processor/mode before testing to ensure consistent audibility across all conditions. Detailed configuration parameters are provided in the Supplementary Table 1.

The primary endpoint of this study was defined as the difference in sentence recognition performance in the S0N0 co-located speech-shaped noise paradigm between the legacy processor (baseline control) and the SONNET 2 omnidirectional (S2.OMNI) configuration. All other speech recognition outcomes (monosyllabic word, disyllabic word, sentence recognition in quiet, and sentence recognition in noise for the S2.Adaptive, R3.OMNI, and R3.Adaptive configurations) were defined as secondary exploratory endpoints. Confirmatory analyses (linear mixed-effects modeling and pre-specified subgroup analyses) were exclusively performed for the primary endpoint, while pairwise comparative analyses were conducted for all secondary endpoints.

### 2.4 Statistical analysis

All statistical analyses were performed using IBM SPSS Statistics version 27.0 and R version 4.5.3. A two-sided significance level (*α*) of 0.05 was adopted for all tests, with *p* < 0.05 considered statistically significant. For all within-subject pairwise comparisons between each upgraded processor configuration and the legacy baseline control, the normality of paired differences was first assessed using the Shapiro–Wilk test. The paired samples *t*-test was applied for normally distributed paired differences, while the non-parametric Wilcoxon signed-rank test was used for non-normally distributed data. To ensure direct comparability of effect sizes across all pairwise comparisons, Cliff’s delta with 95% confidence interval was reported as the unified effect size measure for all analyses, regardless of the statistical test used. Confidence intervals for Cliff’s delta were computed using an independent-samples approach, which may produce slightly wider intervals than the paired-sample tests used for p-values. Statistical significance is determined by the p-values (and FDR-adjusted q-values) from the paired-sample tests (Wilcoxon signed-rank or paired t-test). Negative values of Cliff’s delta indicate higher speech recognition scores in the upgraded processor configuration relative to the legacy baseline. Type I error inflation from multiple pairwise comparisons was controlled using the Benjamini-Hochberg false discovery rate (FDR) procedure, with FDR-adjusted *q*-values reported alongside uncorrected *p*-values.

To quantify the independent effect of audio processor upgrade on the primary endpoint (sentence recognition in S0N0 noise), linear mixed-effects models (LMMs) were constructed exclusively for the comparison between the legacy baseline and the S2.OMNI configuration. To account for within-subject correlation across repeated measurements, a random intercept for each participant was specified in the model. Time point (pre-upgrade legacy processor vs. post-upgrade S2.OMNI configuration), implant type, unilateral versus bilateral implantation status, age at implantation, duration of CI use, legacy processor model, and current age were included as fixed effects. To further explore whether these covariates modulated the treatment effect of processor upgrade, two-way interaction terms between time point and each fixed-effect covariate were introduced into the model in a stepwise hierarchical manner. Nonsignificant interaction terms were stepwise removed from the final model to retain only statistically significant main effects; for any significant interaction identified, post-hoc simple effect analyses were conducted to decompose the interaction effect. Pre-specified subgroup analyses were performed exclusively for the primary endpoint comparison (S2.OMNI vs. legacy processor) to explore participant- and device-related factors associated with speech recognition benefits from processor upgrade.

## 3 Results

### 3.1 Monosyllabic word recognition in quiet

In quiet, the proportion of participants with a monosyllabic word recognition score ≥90% was 1.96% (95% CI: 0.00%–10.40%) for the legacy processor, 7.84% (95% CI: 2.20%–18.90%) for R3.OMNI, 13.73% (95% CI: 5.70%–26.30%) for S2.OMNI, 1.96% (95% CI: 0.00%–10.40%) for R3.Adaptive, and 11.76% (95% CI: 4.40%–23.90%) for the S2.Adaptive configuration (Table 1).

**Table 1:** Word recognition scores (WRS, %) of monosyllabic words, disyllabic words, and sentences in quiet environment.

Speech type	Processor configuration	Score (%), median [IQR]	Proportion of participants with score ≥90% (95% CI)	Comparison with legacy (*p*-value, *q*-value)	Intra-processor mode comparison (*p*-value, *q*-value)	Inter-processor same-mode comparison (S2 vs R3, *p*-value, *q*-value)
Monosyllabic Words	Legacy	65 [45, 70]	1.96% (0.000–0.104)	– (Reference Group)	–	–
	R3.OMNI	70 [50, 80]	7.84% (0.022–0.189)	0.006 (0.012)*	–	0.002 (0.0053) (vs S2. OMNI)*
	R3.Adaptive	70 [55, 80]	1.96%(0.000–0.104)	0.008 (0.0128)†	0.62 (0.62) (vs R3. OMNI)*	0.171 (0.228) (vs S2. Adaptive)*
	S2.OMNI	77.5 [60,85]	13.73% (0.057–0.263)	<0.001 (0.004)*	–	–
	S2.Adaptive	75 [60,80]	11.76% (0.044–0.239)	<0.001 (0.004)*	0.215 (0.246) (vs S2. OMNI)*	–
Disyllabic Words	Legacy	75 [60,80]	11.76% (0.044–0.239)	– (Reference Group)	–	–
	R3.OMNI	80 [75,85]	7.84% (0.022–0.189)	0.004 (0.016)*	–	0.397 (0.529) (vs S2. OMNI)*
	R3.Adaptive	75 [55,80]	13.73% (0.057–0.263)	0.754 (0.754)†	0.028 (0.045) (vs R3. OMNI)†	0.011 (0.029) (vs S2. Adaptive)†
	S2.OMNI	85 [70,90]	27.45% (0.159–0.417)	0.002 (0.016)*	–	–
	S2.Adaptive	75 [70,87.5]	23.53% (0.128–0.375)	0.025 (0.045)†	0.583 (0.666) (vs S2. OMNI)*	–
Sentences	Legacy	85 [60,100]	49.02% (0.348–0.634)	– (Reference Group)	–	–
	R3.OMNI	90 [75,100]	58.82% (0.442–0.724)	0.049 (0.196)*	–	†0.919 (0.930) (vs S2. OMNI)
	R3.Adaptive	80 [70,100]	47.06% (0.329–0.615)	0.470 (0.627)*	0.086 (0.196) (vs R3. OMNI)*	0.137 (0.219) (vs S2. Adaptive)†
	S2.OMNI	90 [80,100]	54.90% (0.403–0.689)	0.067 (0.196)*	–	–
	S2.Adaptive	90 [80,97.5]	50.98% (0.366–0.652)	0.098 (0.196)*	0.930 (0.930) (vs S2. OMNI)*	–

Score (%), median [IQR]: median sentence recognition score with interquartile range for non-normally distributed data. Legacy: Legacy processor; R3: RONDO3 processor; S2: SONNET2 processor; OMNI: omnidirectional mode; Adaptive: Adaptive Intelligence mode.

*Wilcoxon signed-rank test, FDR-adjusted *q*-value in parentheses.

†Paired samples *t*-test, FDR-adjusted *q*-value in parentheses.

Pairwise between-condition comparisons revealed that all four upgraded processor configurations yielded significantly superior monosyllabic word recognition performance relative to the legacy baseline processor (detailed statistical results are summarized in Table 1). Specifically, relative to the legacy processor, significant improvements in recognition performance were observed for the following configurations: R3.OMNI (Cliff’s delta = −0.178, 95% CI [−0.383, 0.044], *p* = 0.006, FDR-adjusted *q* = 0.012), R3.Adaptive (Cliff’s delta = −0.233, 95% CI [−0.436, −0.007], *p* = 0.008, FDR-adjusted *q* = 0.013), S2.OMNI (Cliff’s delta = −0.406, 95% CI [−0.587, −0.186], *p* < 0.001, FDR-adjusted *q* = 0.004), and S2.Adaptive (Cliff’s delta = −0.304, 95% CI [−0.496, −0.084], *p* < 0.001, FDR-adjusted *q* = 0.004).

Intra-processor mode comparisons (omnidirectional vs. adaptive mode within the same processor platform) revealed no statistically significant difference in recognition performance for either the R3 platform (R3.Adaptive vs. R3.OMNI: Cliff’s delta = −0.042, 95% CI [−0.262, 0.182], *p* = 0.62, FDR-adjusted *q* = 0.62) or the S2 platform (S2.Adaptive vs. S2.OMNI: Cliff’s delta = −0.136, 95% CI [−0.351, 0.092], *p* = 0.215, FDR-adjusted *q* = 0.246).

For inter-processor comparisons under matched operational modes, the S2.OMNI configuration significantly outperformed the R3.OMNI configuration (Cliff’s delta = −0.233, 95% CI [−0.438, −0.005], *p* = 0.002, FDR-adjusted *q* = 0.005). In contrast, no statistically significant performance difference was detected between the S2.Adaptive and R3.Adaptive configurations (Cliff’s delta = −0.059, 95% CI [−0.273, 0.160], *p* = 0.171, FDR-adjusted *q* = 0.228).

### 3.2 Disyllabic word recognition in quiet

In quiet, the proportion of participants with a disyllabic word recognition score ≥90% was 11.76% (95% CI: 4.40%–23.90%) for the legacy processor, 7.84% (95% CI: 2.20%–18.90%) for R3.OMNI, 27.45% (95% CI: 15.90%–41.70%) for S2.OMNI, 13.73% (95% CI: 5.70%–26.30%) for R3.Adaptive, and 23.53% (95% CI: 12.80%–37.50%) for the S2.Adaptive configuration (Table 1).

Pairwise between-condition comparisons revealed significant differences in disyllabic word recognition performance between the upgraded processor configurations and the legacy baseline processor, with full statistical results summarized in Table 1. Specifically, relative to the legacy processor, significantly superior recognition performance was observed for three of the four upgraded configurations: R3.OMNI (Cliff’s delta = −0.233, 95% CI [−0.438, −0.005], *p* = 0.004, FDR-adjusted *q* = 0.016), S2.OMNI (Cliff’ s delta = −0.272, 95% CI [−0.472, −0.046], *p* = 0.002, FDR-adjusted *q* = 0.016), and S2.Adaptive (Cliff’s delta = −0.191, 95% CI [−0.400, 0.036], *p* = 0.025, FDR-adjusted *q* = 0.045). In contrast, no statistically significant performance difference was detected between the R3.Adaptive configuration and the legacy processor (Cliff’s delta = −0.043, 95% CI [−0.263, 0.181], *p* = 0.754, FDR-adjusted *q* = 0.754).

Intra-processor mode comparisons (omnidirectional vs. adaptive mode within the same processor platform) revealed that adaptive mode significantly outperformed omnidirectional mode for the R3 platform (R3.Adaptive vs. R3.OMNI: Cliff’s delta = 0.163, 95% CI [−0.066, 0.375], *p* = 0.028, FDR-adjusted *q* = 0.045), while no statistically significant difference was detected for the S2 platform the S2 platform (S2.Adaptive vs. S2.OMNI: Cliff’s delta = −0.062, 95% CI [−0.282, 0.164], *p* = 0.583, FDR-adjusted *q* = 0.666).

For inter-processor comparisons under matched operational modes, the S2.Adaptive configuration significantly outperformed the R3.Adaptive configuration (*p* = 0.011, FDR-adjusted *q* = 0.029), while no statistically significant performance difference was identified between the S2.OMNI and R3.OMNI configurations (*p* = 0.397, FDR-adjusted *q* = 0.529).

### 3.3 Sentence recognition in quiet

In quiet, the proportion of participants with a sentence recognition score ≥90% was 49.02% (95% CI: 34.80%–63.40%) for the legacy processor, 58.82% (95% CI: 44.20%–72.40%) for R3.OMNI, 54.90% (95% CI: 40.30%–68.90%) for S2.OMNI, 47.06% (95% CI: 32.90–61.50%) for R3.Adaptive, and 50.98% (95% CI: 36.60–65.20%) for the S2.Adaptive configuration (Table 1).

Pairwise between-condition comparisons revealed no statistically significant differences in sentence recognition performance between any of the upgraded processor configurations and the legacy baseline processor after FDR correction, with full statistical results summarized in Table 1. Specifically, the R3.OMNI configuration showed a borderline statistically significant improvement in sentence recognition relative to the legacy processor in uncorrected analyses (Cliff’s delta = −0.129, 95% CI [−0.341, 0.096], uncorrected *p* = 0.049, FDR-adjusted *q* = 0.196), but this effect did not survive multiple comparison correction. No statistically significant performance differences were detected between the legacy processor and the remaining three upgraded configurations: R3.Adaptive (Cliff’s delta = −0.008, 95% CI [−0.231, 0.215], *p* = 0.470, FDR-adjusted *q* = 0.627), S2.OMNI (Cliff’s delta = −0.109, 95% CI [−0.322, 0.114], *p* = 0.067, FDR-adjusted *q* = 0.196), and S2.Adaptive (Cliff’s delta = −0.072, 95% CI [−0.289, 0.152], *p* = 0.098, FDR-adjusted *q* = 0.196).

Intra-processor mode comparisons (omnidirectional vs. adaptive mode within the same processor platform) revealed no statistically significant differences in sentence recognition performance for either the R3 platform (R3.Adaptive vs. R3.OMNI: *p* = 0.086, FDR-adjusted *q* = 0.196) or the S2 platform (S2.Adaptive vs. S2.OMNI: *p* = 0.930, FDR-adjusted *q* = 0.930). Similarly, inter-processor comparisons under matched operational modes revealed no significant between-platform performance differences in quiet (R3.OMNI vs. S2.OMNI: *p* = 0.919, FDR-adjusted *q* = 0.930; R3.Adaptive vs. S2.Adaptive: *p* = 0.137, FDR-adjusted *q* = 0.219).

### 3.4 Spatially co-located speech and noise (S0N0 test paradigm)

In the S0N0 test condition (speech and noise presented at 0° azimuth in the horizontal plane), the proportion of participants with a sentence recognition score ≥90% was 43.14% (95% CI: 29.30%–57.80%) for the legacy processor, 49.02% (95% CI: 34.80%–63.40%) for R3.OMNI, 52.94% (95% CI: 38.50%–67.10%) for S2.OMNI, 43.14% (95% CI: 29.30%–57.80%) for R3.Adaptive, and 49.02% (95% CI: 34.80%–63.40%) for the S2.Adaptive configuration (Table 2).

**Table 2:** Sentence recognition scores (SRS, %) across different noise directions.

Noise direction	Processor configuration	Score (%), median [IQR]	Proportion of participants with score ≥90% (95% CI)	Comparison with legacy (*p*-value, *q*-value)	Intra-processor mode comparison (*p*-value, *q*-value)	Inter-processor same-mode comparison (S2 vs R3, *p*-value, *q*-value)
0°, S0N0	Legacy	82.5 [60,90]	43.14% (0.293–0.578)	– (Reference Group)	–	–
	R3.OMNI	87.5 [70,95]	49.02% (0.348–0.634)	0.011 (0.044)*	–	0.458 (0.458) (vs S2. OMNI)*
	R3.Adaptive	85 [70,95]	43.14% (0.293–0.578)	0.165 (0.264)†	0.104 (0.208) (vs R3. OMNI)*	0.458 (0.458) (vs S2. Adaptive)*
	S2.OMNI	90 [80,97.5]	52.94% (0.385–0.671)	<0.001 (0.008)*	–	–
	S2.Adaptive	85 [70,95]	49.02% (0.348–0.634)	0.078 (0.208)*	0.199 (0.265) (vs S2. OMNI)*	–

Score (%), median [IQR]: median sentence recognition score with interquartile range for non-normally distributed data. Legacy: Legacy processor; R3: RONDO3 processor; S2: SONNET2 processor; OMNI: omnidirectional mode; Adaptive: Adaptive Intelligence mode.

*Wilcoxon signed-rank test, FDR-adjusted *q*-value in parentheses.

†Paired samples *t*-test, FDR-adjusted *q*-value in parentheses.

Pairwise between-condition comparisons revealed significant differences in sentence recognition performance in noise between the upgraded processor configurations and the legacy baseline processor, with full statistical results summarized in Table 2. Specifically, relative to the legacy processor, significantly superior recognition performance was observed for two of the four upgraded configurations: R3.OMNI (Cliff’s delta = −0.141, 95% CI [−0.353, 0.084], *p* = 0.011, FDR-adjusted *q* = 0.044) and S2.OMNI (Cliff’s delta = −0.193, 95% CI [−0.400, 0.032], *p* < 0.001, FDR-adjusted *q* = 0.008). In contrast, no statistically significant performance differences were detected between the legacy processor and the remaining two upgraded configurations after FDR correction: R3.Adaptive (Cliff’s delta = −0.057, 95% CI [−0.276, 0.168], *p* = 0.165, FDR-adjusted *q* = 0.264) and S2.Adaptive (Cliff’s delta = −0.107, 95% CI [−0.322, 0.118], *p* = 0.078, FDR-adjusted *q* = 0.208).

Intra-processor mode comparisons (omnidirectional vs. adaptive mode within the same processor platform) revealed no statistically significant differences in sentence recognition performance for either the R3 platform (R3.Adaptive vs. R3.OMNI: Cliff’s delta = 0.082, 95% CI [−0.143, 0.299], *p* = 0.104, FDR-adjusted *q* = 0.208) or the S2 platform (S2.Adaptive vs. S2.OMNI: Cliff’s delta = 0.120, 95% CI [−0.106, 0.334], *p* = 0.199, FDR-adjusted *q* = 0.265). Similarly, inter-processor comparisons under matched operational modes revealed no significant between-platform performance differences in the S0N0 noise condition (R3.OMNI vs. S2.OMNI: *p* = 0.458, FDR-adjusted *q* = 0.458; R3.Adaptive vs. S2.Adaptive: *p* = 0.458, FDR-adjusted *q* = 0.458).

### 3.5 Linear mixed-effects model (LMM) analysis

LMM Analysis for the Primary Endpoint To quantify the independent effect of audio processor upgrade on the pre-specified primary endpoint (sentence recognition performance in the S0N0 noise paradigm), a LMM was constructed for the core comparison between the legacy baseline processor and the upgraded S2.OMNI configuration. After adjusting for pre-specified fixed-effect covariates, including implantation status (unilateral vs. bilateral), implant type, age at implantation, duration of CI use, and current participant age, upgrade to the S2.OMNI configuration was found to exert a statistically significant positive effect on sentence recognition performance in noise (*F* = 9.885, *p* = 0.003). None of the included covariates exerted a statistically significant independent effect on recognition outcomes: implantation status (unilateral vs. bilateral, *F* = 1.222, *p* = 0.276), implant type (*F* = 1.046, *p* = 0.396), age at implantation (*F* = 0.588, *p* = 0.448), duration of CI use (*F* = 1.615, *p* = 0.211), and current participant age (*F* = 0.452, *p* = 0.505) (Table 3). Stepwise hierarchical testing was performed for two-way interaction terms between time point and each fixed-effect covariate (implant type, unilateral versus bilateral implantation status, age at implantation, duration of CI use, and current participant age). None of the interaction terms were statistically significant (all *p* > 0.05), suggesting that these factors did not significantly moderate the change in sentence recognition performance associated with audio processor upgrade. Accordingly, the final parsimonious main effects model without interaction terms was used for all confirmatory analyses of the primary endpoint.

**Table 3:** Linear mixed model results for the effect of changing external processor on speech recognition.

Fixed Effect	Numerator df	Denominator df	F	*p*-value
Intercept	1	40	37.117	<0.001
Time point (Pre vs. Post)	1	50	9.885	0.003
Implant type	4	40	1.046	0.396
Legacy processor model	2	40	1.275	0.291
Unilateral/Bilateral	1	40	1.222	0.276
Age at surgery	1	40	0.588	0.448
Duration of use	1	40	1.615	0.211
Age	1	40	0.452	0.505

### 3.6 Subgroup analysis

**Figure 1:**
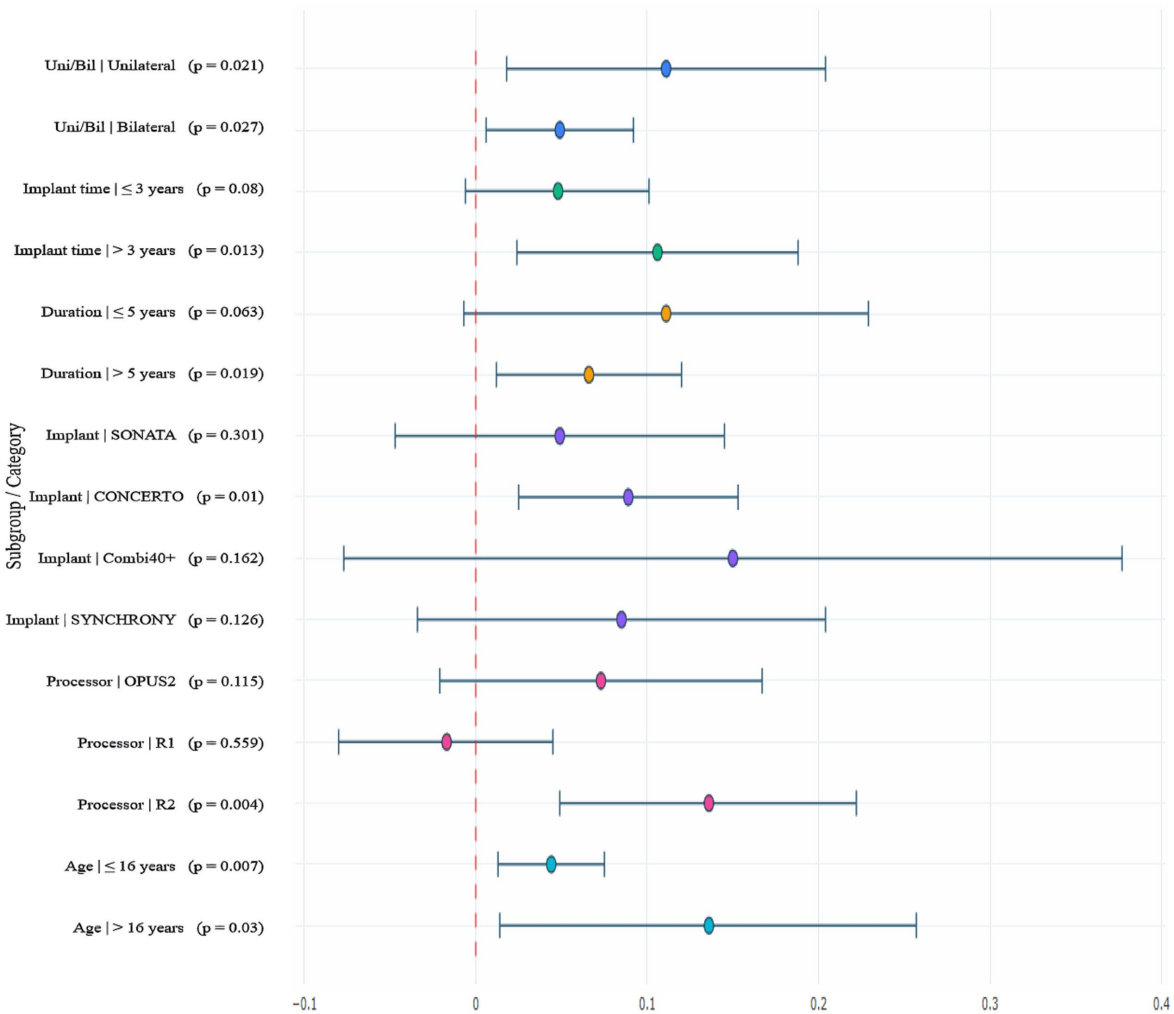
This forest plot shows pre-specified subgroup analyses for the study’s primary endpoint: the change in sentence recognition score in the SONO co-located speech-shaped noise paradigm, comparing the SONNET 2 omnidirectional (S2.OMNI) configuration to the legacy baseline processor in native Mandarin-speaking cochlear implant users. Positive values indicate improved speech recognition with S2.OMNI. Solid circles represent the point estimate of score change for each subgroup, horizontal lines represent the corresponding 95% confidence intervals, and the vertical solid line at 0 marks no between-configuration performance difference. *p*-values for each subgroup correspond to within-subject pairwise comparisons, with *p* < 0.05 considered statistically significant. SONO, speech and noise presented at 0° azimuth; OMNI, omnidirectional mode.

Pre-specified Subgroup Analysis for the Primary Endpoint To further explore participant- and device-related factors associated with speech recognition benefits from upgrade to the S2.OMNI configuration, pre-specified subgroup analyses were performed based on the primary endpoint comparison (S2.OMNI vs. legacy baseline processor) in the S0N0 noise test condition. Results for each subgroup are detailed in Table 4 and Figure 1.

**Table 4:** Comparisons of word recognition scores (WRS) in S0N0.

Subgroup variable	Category	Difference (Change)	95% CI	*p*-value
Unilateral/bilateral	Unilateral	0.111	0.018–0.204	0.021
	Bilateral	0.049	0.006–0.092	0.027
Time since implantation	≤3 years	0.048	−0.006–0.101	0.08
	>3 years	0.106	0.024–0.188	0.013
Duration of use	≤5 years	0.111	−0.007–0.229	0.063
	>5 years	0.066	0.012–0.120	0.019
Implant type	SONATA	0.049	−0.047–0.145	0.301
	CONCERTO	0.089	0.025–0.153	0.01
	Combi40+	0.15	−0.077–0.377	0.162
	SYNCHRONY	0.085	−0.034–0.204	0.126
Legacy processor type	OPUS2	0.073	−0.021–0.167	0.115
	R1	−0.017	−0.080–0.045	0.559
	R2	0.136	0.049–0.222	0.004
Age	≤16 years	0.044	0.013–0.075	0.007
	>16 years	0.136	0.014–0.257	0.03

Implantation Laterality Both unilateral (change in sentence recognition score = 0.111, 95% CI: 0.018–0.204, *p* = 0.021) and bilateral CI users (change in score = 0.049, 95% CI: 0.006–0.092, *p* = 0.027) achieved statistically significant improvements in recognition performance following upgrade to the S2.OMNI configuration.

Implantation Duration Participants with an implantation duration >3 years (i.e., >3 years of CI use) showed a statistically significant improvement in sentence recognition performance (change in score = 0.106, 95% CI: 0.024–0.188, *p* = 0.013) after upgrade to the S2.OMNI configuration. In contrast, no statistically significant improvement was observed in participants with an implantation duration ≤3 years (change in score = 0.048, 95% CI: −0.006–0.101, *p* = 0.080).

Legacy Processor Usage Duration Participants who used the legacy processor for >5 years obtained a statistically significant speech recognition benefit from upgrade to the S2.OMNI configuration (change in score = 0.066, 95% CI: 0.012–0.120, *p* = 0.019). Conversely, no statistically significant improvement was detected in participants with a legacy processor usage duration ≤5 years (change in score = 0.111, 95% CI: −0.007–0.229, *p* = 0.063).

Implant Type Statistically significant performance improvement was observed in participants with the CONCERTO implant after upgrade to the S2.OMNI configuration (change in score = 0.089, 95% CI: 0.025–0.153, *p* = 0.010). No statistically significant changes in recognition performance were found in users of the SONATA, Combi40+, or SYNCHRONY implants (all *p* > 0.05).

Participants who had originally used the RONDO 2 legacy processor achieved a statistically significant improvement in sentence recognition after upgrade to the S2.OMNI configuration (change in score = 0.136, 95% CI: 0.049–0.222, *p* = 0.004). No statistically significant benefit was detected in participants using the OPUS2 or RONDO 1 legacy processors (all *p* > 0.05).

Age Group Consistent with the age stratification defined in the Methods section, both pediatric participants aged ≤16 years (change in score = 0.044, 95% CI: 0.013–0.075, *p* = 0.007) and adult participants aged >16 years (change in score = 0.136, 95% CI: 0.014–0.257, *p* = 0.030) obtained statistically significant speech recognition benefits from upgrade to the S2.OMNI configuration.

## 4 Discussion

### 4.1 Principal findings

The primary objective of this prospective single-subject repeated-measures study was to systematically evaluate speech recognition performance of two next-generation dual-microphone audio processors (SONNET 2 [S2] and RONDO 3 [R3], MED-EL) in native Mandarin-speaking CI users, both in quiet and in spatially co-located speech-shaped noise (S0N0 test paradigm). The principal findings of the study are synthesized below, aligned with the pre-specified primary and secondary endpoints: For the pre-specified primary endpoint, confirmatory analyses demonstrated that upgrade to the S2.OMNI configuration significantly enhanced sentence recognition in the S0N0 noise paradigm relative to the legacy baseline processor. LMM analysis confirmed that upgrade to the S2.OMNI configuration exerted a statistically significant independent positive effect on sentence recognition performance in noise, with no significant confounding effects from implantation laterality, implant type, age at implantation, duration of CI use, or current participant age. Pre-specified subgroup analyses further demonstrated consistent significant benefits from upgrade to the S2.OMNI configuration across pediatric and adult users, as well as unilateral and bilateral CI recipients. More pronounced benefits were observed in specific subgroups, including users with >3 years of CI use, >5 years of legacy processor usage, those implanted with the CONCERTO device, and those originally using the RONDO 2 legacy processor. For the secondary exploratory endpoints, pairwise comparative analyses yielded the following key findings: 1. In quiet environments, all four upgraded processor configurations (S2.OMNI, S2.Adaptive, R3.OMNI, R3.Adaptive) yielded significantly superior monosyllabic word recognition performance relative to the legacy baseline processor, regardless of operational mode. For disyllabic word recognition, all upgraded configurations except R3.Adaptive also showed statistically significant improvements over the legacy device. No statistically significant between-group differences in sentence recognition in quiet were observed for any upgraded configuration after FDR correction. 2. In the S0N0 noise condition, the R3.OMNI configuration also significantly enhanced sentence recognition relative to the legacy processor in pairwise analyses, while no statistically significant benefit was observed for the adaptive intelligence (Adaptive) modes of either processor after FDR correction. This study is the first large-scale prospective investigation to evaluate the clinical performance of these two dual-microphone audio processors in a native Mandarin-speaking CI population. Unlike non-tonal Indo-European languages, Mandarin Chinese is a tonal language in which pitch contours are critical for distinguishing lexical meaning, creating unique signal processing challenges for CI devices. Our findings fill a critical gap in the existing literature, which has predominantly focused on non-tonal language speakers, and provide evidence to guide clinical device selection and personalized upgrade decision-making for Mandarin-speaking CI users.

### 4.2 Performance in quiet environments and alignment with existing literature

Our results demonstrated that the S2 and R3 processors delivered significant improvements in monosyllabic and disyllabic word recognition in quiet, which aligns with previous studies showing that audio processor upgrades can enhance speech perception performance in CI users (Weissgerber et al., 2019). However, a unique strength of our study is its targeted focus on tonal language speech recognition. In Mandarin Chinese, monosyllabic words serve as the fundamental lexical units, and accurate identification relies heavily on the precise transmission of fine spectral and temporal cues, including pitch contours required for tone discrimination. The significant improvement in monosyllabic recognition observed here indicates that, compared with legacy devices, the next-generation dual-microphone processors have a superior ability to encode these fine acoustic cues critical for Mandarin speech understanding.

In contrast to word-level recognition, no statistically significant improvement in sentence recognition was observed in quiet between the upgraded and legacy processors after FDR correction. This finding is consistent with previous reports that CI users generally achieve high baseline levels of sentence recognition in quiet, resulting in a ceiling effect that limits the ability to detect incremental benefits from device upgrades. Unlike word recognition tasks, sentence recognition is supported by rich contextual and semantic cues, which allow listeners to compensate for deficits in phonemic or syllabic identification. Monosyllabic and disyllabic word recognition tasks, by contrast, lack contextual support, making them more sensitive, objective measures of the intrinsic signal processing performance of an audio processor.

For inter-device comparisons in quiet, S2.OMNI significantly outperformed R3.OMNI in monosyllabic word recognition. This performance difference is most likely attributable to the distinct form factors of the two devices. The S2 is a behind-the-ear (BTE) processor with microphones positioned near the physiological pinna, which provides natural acoustic amplification and superior high-frequency signal pickup. In contrast, the R3 is an OTE processor mounted on the mastoid, where the pinna’s natural acoustic gain effect is attenuated.

For intra-device mode comparisons in quiet, we found no statistically significant difference between OMNI and Adaptive modes for monosyllabic word and sentence recognition, with the exception of disyllabic word recognition, where the R3.Adaptive mode significantly outperformed the R3.OMNI mode for both devices. This finding aligns with the core design principle of the adaptive intelligence system, which is optimized for complex noise environments rather than quiet conditions. In quiet, the adaptive intelligence system automatically disables noise reduction and beamforming algorithms, maintaining only baseline signal processing, which results in minimal performance differences between modes. The small but significant benefit of Adaptive mode in disyllabic recognition may be attributed to the system’s optimization of weak acoustic cues, such as neutral tones and inter-syllabic transition cues, which are critical for accurate disyllabic word identification in Mandarin. These results are consistent with the findings of Kurz et al. (2022), who reported minimal performance differences between Adaptive and OMNI modes in quiet environments for the S2 processor.

### 4.3 Speech recognition in noise: mechanistic interpretations

Impaired speech recognition in background noise remains the primary functional limitation for CI users, and thus improving noise robustness is the core goal of modern CI signal processing development. Our study found that the OMNI modes of both S2 and R3 significantly improved sentence recognition in the S0N0 noise condition relative to the legacy processor. This benefit is primarily driven by hardware-level advancements in the next-generation processors: the dual-microphone array features improved pre-amplification circuits, higher analog-to-digital conversion precision, and a higher intrinsic signal-to-noise ratio (SNR) for sound pickup, even without activation of adaptive beamforming and noise reduction algorithms (Carlyon and Goehring, 2021; Borjigin et al., 2024; Tahmasebi et al., 2020). These hardware upgrades enhance the fidelity of original acoustic signal acquisition, which directly translates to improved speech recognition in noise. Our findings are consistent with those of Honeder et al. (2018), who demonstrated that next-generation dual-microphone arrays provide fundamental improvements in signal acquisition performance, even in omnidirectional mode.

Notably, we did not observe a statistically significant benefit of the Adaptive modes relative to the legacy processor in the S0N0 paradigm after FDR correction. This finding can be explained by three key factors related to our study design and the mechanistic basis of adaptive signal processing. First, the adaptive beamforming (directional microphone) system, a core component of the adaptive intelligence algorithm, achieves SNR improvement by spatially separating target speech and background noise: it enhances signals from the front while suppressing noise from the sides and rear. In our S0N0 paradigm, both speech and noise were presented from the same 0° azimuth, eliminating the spatial contrast required for beamforming to exert its effect. Previous studies have consistently shown that adaptive directional systems provide the greatest benefit when target speech and noise are spatially separated (Weissgerber et al., 2019; Kuk et al., 2023), and our results confirm that this advantage is not apparent in co-located noise conditions. Second, our study employed an acute testing paradigm, with no long-term acclimation period for the Adaptive mode. Previous research has demonstrated that CI users require several weeks of acclimation to fully adapt to and optimize the benefit of adaptive noise reduction algorithms (Hey et al., 2022), meaning acute testing may underestimate the real-world clinical benefit of the adaptive intelligence system. Third, the 10 dB SNR used in our study (70 dB SPL speech vs. 60 dB SPL noise) may have resulted in a partial ceiling effect: 43.14% of participants already achieved sentence recognition scores ≥90% with the legacy processor, limiting the room for further improvement with adaptive algorithms.

For inter-device comparisons in noise, we found no statistically significant difference between S2 and R3 under matched operational modes, indicating that the two processors have equivalent core signal processing algorithms and dual-microphone array performance, despite their different form factors. This finding aligns with existing literature on dual-microphone CI processor performance. Wimmer et al. (2015) previously observed inferior speech perception with OTE processors compared to BTE processors in the S0N180 speech-noise configuration, a discrepancy ascribed to the more posterior microphone position of OTE devices. However, that study employed legacy single-microphone processors, which cannot leverage microphone directionality. By contrast, Mauger et al. (2017) demonstrated that dual-microphone technology mitigates most performance variations associated with differences in microphone positioning between OTE and BTE processors. Our results extend these findings, showing equivalent noise performance between BTE and OTE dual-microphone processors even in co-located noise conditions. This is a clinically impactful finding: the R3 OTE processor offers significant advantages in terms of wearing convenience, wireless charging, and no requirement for daily drying, while maintaining non-inferior speech recognition performance in noise compared with the BTE S2 processor.

### 4.4 Clinical implications of linear mixed-effects model and subgroup analyses

Our LMM analysis confirmed that audio processor upgrade exerted a statistically significant independent positive effect on sentence recognition performance in noise (*F* = 9.885, *p* = 0.003), with no significant confounding effects from implantation laterality, implant type, age at implantation, duration of CI use, or current participant age. This is a clinically impactful finding for routine practice: it demonstrates that CI users can achieve significant speech perception benefits from upgrading the external audio processor, regardless of baseline implantation characteristics, and without the need for invasive revision surgery. This finding aligns with Mosnier et al. (2021), who reported significant benefits from processor upgrade in CI users with more than 20 years of implant experience, and supports the routine consideration of processor upgrade for long-term CI users during clinical follow-up.

Our prespecified subgroup analyses further refined the clinical translation of our findings, identifying patient subgroups that derive the greatest magnitude of benefit from processor upgrade. First, significant upgrade-related benefits were observed in both pediatric (≤16 years) and adult (>16 years) CI users. Most prior studies of processor upgrade have focused on post-lingually deafened adult users, and our study is among the first to validate the efficacy of next-generation CI processors in a large cohort of native Mandarin-speaking pediatric CI users. For children in the critical period of speech and language development, improved monosyllabic word recognition and speech-in-noise performance have profound implications for spoken language acquisition, academic attainment, and long-term social development.

Second, significant post-upgrade improvements were seen in both unilateral and bilateral CI recipients, indicating that the benefit of processor upgrade is not dependent on binaural integration capacity. This finding is particularly relevant for the large global population of unilateral CI users, for whom processor upgrade represents a low-risk, high-yield intervention to enhance functional hearing performance without additional surgery.

Third, the most pronounced benefits from upgrade were observed in users with >3 years of CI experience and >5 years of legacy processor use. This finding is biologically and clinically intuitive: longer-term use of older-generation devices creates a wider performance gap between legacy and next-generation processors, translating to more substantial incremental benefits from upgrade. These results suggest that clinicians should prioritize processor upgrade counselling for CI users who have used legacy devices for 5 years or more, to maximize functional hearing outcomes.

Finally, the greatest upgrade-related benefits were seen in users with CONCERTO implants and those originally using the RONDO 2 legacy processor. This is most likely attributable to higher hardware compatibility and optimized signal transmission protocols between the newer-generation CONCERTO implant and the S2/R3 processors, as well as the larger generational gap in signal processing algorithms between the RONDO 2 and the next-generation devices. These findings can directly guide clinicians in developing personalized upgrade recommendations tailored to a patient’s existing implant and legacy processor model.

### 4.5 Study limitations

Several key limitations of this study must be acknowledged when interpreting the findings. First, speech recognition testing was only performed using speech-shaped noise (SSN) in a single spatial configuration (S0N0 test paradigm). We did not evaluate more ecologically valid noise types commonly encountered in daily life, including multi-talker babble, transient environmental noise, and wind noise; nor did we assess spatially separated speech-noise configurations where adaptive beamforming algorithms exert their maximal effect. This means our study may have underestimated the real-world functional benefit of the AI system.

Second, this study employed an acute in-laboratory testing paradigm, with no long-term acclimation period for the new processors or operational modes. As noted in prior sections, CI users typically require several weeks of device acclimation to fully adapt to and optimize the benefit of adaptive signal processing algorithms (Hey et al., 2022). Our acute testing design may therefore have underestimated the sustained long-term clinical benefit of the Adaptive mode.

Third, the 10 dB signal-to-noise ratio (SNR) used for noise testing may have introduced a partial ceiling effect, limiting our ability to detect more nuanced performance differences between processor configurations. Future studies using lower, more challenging SNRs will be better equipped to characterize the performance limits of different signal processing strategies.

Fourth, due to constraints on total testing time, we only evaluated the OMNI and Adaptive modes, and did not assess fixed beamforming or other directional microphone modes available on the devices. This prevents a comprehensive head-to-head comparison of all available signal processing strategies in these next-generation processors.

Finally, we did not implement a strictly randomized, balanced test order, instead using a fixed test sequence for all participants. While we employed randomized, non-repeating speech test lists to minimize word memorization and learning effects, we cannot completely exclude the potential influence of incremental learning on our study results.

### 4.6 Future research directions

Future investigations should address the key limitations outlined above and extend the clinical and scientific insights of this work. First, studies should incorporate more ecologically valid listening scenarios, including multi-talker babble, transient environmental noise, wind noise, and spatially separated speech-noise configurations, to comprehensively evaluate the real-world performance of the adaptive intelligence system in daily listening environments. Second, long-term prospective follow-up studies are needed to assess the sustained speech recognition benefits of the new processors after 1–3 months of real-world acclimation, as well as their impact on patient-reported health-related quality of life. Third, further research should specifically investigate the effect of processor upgrade on Mandarin lexical tone recognition, to clarify the mechanistic pathways by which next-generation processors optimize the encoding of tonal cues that are critical for Mandarin speech understanding. Finally, comprehensive assessments of spatial localization ability, head shadow effects, and binaural hearing performance will further complete the clinical performance profile of these two next-generation dual-microphone processors.

## 5 Conclusion

This prospective single-subject repeated-measures study provides confirmatory evidence that upgrade to the SONNET 2 omnidirectional (S2.OMNI) dual-microphone audio processor configuration significantly improves sentence recognition in co-located speech-shaped noise in native Mandarin-speaking CI users. Audio processor upgrade to the S2.OMNI configuration is an independent predictor of improved speech recognition performance in noise, with consistent significant benefits across pediatric and adult users, as well as unilateral and bilateral CI users. For secondary exploratory outcomes, the SONNET 2 and RONDO 3 dual-microphone audio processors significantly improve monosyllabic and disyllabic word recognition in quiet, and the RONDO 3 omnidirectional configuration also significantly improves sentence recognition in co-located speech-shaped noise, in native Mandarin-speaking CI users. These findings fill a critical gap in the existing CI literature, which has predominantly focused on non-tonal language populations, and provide high-level evidence to guide clinical device selection and personalized upgrade decision-making for Mandarin-speaking CI users.

## Data Availability

The raw data supporting the conclusions of this article will be made available by the authors, without undue reservation.
