# Supplementary material for: Speech recognition performance with dual-microphone audio processors in mandarin-speaking cochlear implant users
Source: Front Neurosci. 2026 Apr 23;20:1767325. doi: 10.3389/fnins.2026.1767325 (PMC13149284; doi:10.3389/fnins.2026.1767325)
Supplement: Supplementary file 1 [file Table_1.DOCX]

Supplementary Table 1. Signal processing parameter settings of all tested audio processor configurations

| **Audio Processor** | **Configuration** | **AI** | **WNR** | **Direct.** | **ANR** | **TNR** |
| --- | --- | --- | --- | --- | --- | --- |
| SONNET 2 | S2.AImild (clinical default) | Mild | Mild | Auto (NAT) | Auto (Off-Mild) | Auto (Off-Mild) |
| SONNET 2 | SONNET 2 OMNI | Off | Mild | OMNI | N/A | N/A |
| R3 | R3.AImild (clinical default) | Mild | Mild | Auto (NAT) | Auto (Off-Mild) | Auto (Off-Mild) |
| R3 | R3OMNI | Off | Mild | OMNI | N/A | N/A |
| Opus2 | (clinical default) | N/A | N/A | OMNI | N/A | N/A |
| RONDO | (clinical default) | N/A | N/A | OMNI | N/A | N/A |
| RONDO2 | (clinical default) | N/A | N/A | OMNI | N/A | N/A |

AI = Adaptive Intelligence; WNR = Wind Noise Reduction; Direct. = Directionality; ANR = Adaptive Noise Reduction; TNR = Transient Noise Reduction; NAT = Natural Adaptive Directionality; OMNI = Omnidirectional; N/A = Not Applicable.
